# Increasing self-efficacy – the effectiveness of a pain management programme for children and parents

**DOI:** 10.1186/1546-0096-9-S1-O43

**Published:** 2011-09-14

**Authors:** A Morgan, N Levi, C Bernie

**Affiliations:** 1 Great Ormond Street Hospital, London, UK

## Background

CBT-based pain management groups have been well documented in the literature to effect functional changes in children and adolescents. Few, however, have examined their capacity to increase a parent’s perceived ability to manage their child’s pain. This is important when considering the emotional and practical influences that parents have upon their children. This study reviews the effectiveness of a brief pain management group comprised of simultaneous parent and child sessions on their perceived ability to cope with pain.

## Aim

The aim of this study was to evaluate the effectiveness of a group programme designed to improve a family’s self efficacy around coping with pain. It was hoped that school attendance would also improve.

## Methods

Parents and children attended three intervention sessions and one 6-week follow-up. Sessions focused on cognitive-behavioural pain management techniques, associated mood management and increasing parental competence to manage pain related behaviour. Pre, Post and Follow Up data was collected from 30 patients over three groups. A Coping VAS and the Paediatric Quality of Life Scale (PEDS-QOL) were used to assess the effectiveness of the group intervention.

## Results

Following intervention, there was a significant increase in the parents’ and child’s perceived ability to cope with pain (t(17)=2.587, p<.05). Social functioning also improved significantly over the course of the intervention according to parent and child report. Psychosocial health was rated as improved by all participants; however given the small number of returned questionnaires this did not reach significance. There was a trend towards increased weekly school attendance. It is hoped these trends will reach significance as more data is collected.

## Conclusions

These findings indicate that a brief pain management group can increase parents’ and children’s self-efficacy to manage pain, and is likely to decrease pain-associated distress. This has important implications as to the management of patients in busy outpatient services; the amount of health visits utilised by families; and the number of days off school as a result of pain.

